# Supplementary material for: Named entity recognition of pharmacokinetic parameters in the scientific literature
Source: Sci Rep. 2024 Oct 8;14:23485. doi: 10.1038/s41598-024-73338-3 (PMC11461509; doi:10.1038/s41598-024-73338-3)
Supplement: Supplementary file 1 — Supplementary Information. [file 41598_2024_73338_MOESM1_ESM.zip › _archive/template.pdf]

A TEMPLATE FOR THE *arxiv* STYLE

A PREPRINT

David S. Hippocampus*
Department of Computer Science
Cranberry-Lemon University
Pittsburgh, PA 15213
`hippo@cs.cranberry-lemon.edu`

Elias D. Striatum
Department of Electrical Engineering
Mount-Sheikh University
Santa Narimana, Levand
`stariate@ee.mount-sheikh.edu`

September 29, 2020

ABSTRACT

Lorem ipsum dolor sit amet, consectetuer adipiscing elit. Ut purus elit, vestibulum ut, placerat ac, adipiscing vitae, felis. Curabitur dictum gravida mauris. Nam arcu libero, nonummy eget, consectetuer id, vulputate a, magna. Donec vehicula augue eu neque. Pellentesque habitant morbi tristique senectus et netus et malesuada fames ac turpis egestas. Mauris ut leo. Cras viverra metus rhoncus sem. Nulla et lectus vestibulum urna fringilla ultrices. Phasellus eu tellus sit amet tortor gravida placerat. Integer sapien est, iaculis in, pretium quis, viverra ac, nunc. Praesent eget sem vel leo ultrices bibendum. Aenean faucibus. Morbi dolor nulla, malesuada eu, pulvinar at, mollis ac, nulla. Curabitur auctor semper nulla. Donec varius orci eget risus. Duis nibh mi, congue eu, accumsan eleifend, sagittis quis, diam. Duis eget orci sit amet orci dignissim rutrum.

Keywords First keyword · Second keyword · More

1 Introduction

Nam dui ligula, fringilla a, euismod sodales, sollicitudin vel, wisi. Morbi auctor lorem non justo. Nam lacus libero, pretium at, lobortis vitae, ultricies et, tellus. Donec aliquet, tortor sed accumsan bibendum, erat ligula aliquet magna, vitae ornare odio metus a mi. Morbi ac orci et nisl hendrerit mollis. Suspendisse ut massa. Cras nec ante. Pellentesque a nulla. Cum sociis natoque penatibus et magnis dis parturient montes, nascetur ridiculus mus. Aliquam tincidunt urna. Nulla ullamcorper vestibulum turpis. Pellentesque cursus luctus mauris. Nulla malesuada porttitor diam. Donec felis erat, congue non, volutpat at, tincidunt tristique, libero. Vivamus viverra fermentum felis. Donec nonummy pellentesque ante. Phasellus adipiscing semper elit. Proin fermentum massa ac quam. Sed diam turpis, molestie vitae, placerat a, molestie nec, leo. Maecenas lacinia. Nam ipsum ligula, eleifend at, accumsan nec, suscipit a, ipsum. Morbi blandit ligula feugiat magna. Nunc eleifend consequat lorem. Sed lacinia nulla vitae enim. Pellentesque tincidunt purus vel magna. Integer non enim. Praesent euismod nunc eu purus. Donec bibendum quam in tellus. Nullam cursus pulvinar lectus. Donec et mi. Nam vulputate metus eu enim. Vestibulum pellentesque felis eu massa.

2 Headings: first level

Quisque ullamcorper placerat ipsum. Cras nibh. Morbi vel justo vitae lacus tincidunt ultrices. Lorem ipsum dolor sit amet, consectetuer adipiscing elit. In hac habitasse platea dictumst. Integer tempus convallis augue. Etiam facilisis. Nunc elementum fermentum wisi. Aenean placerat. Ut imperdiet, enim sed gravida sollicitudin, felis odio placerat quam, ac pulvinar elit purus eget enim. Nunc vitae tortor. Proin tempus nibh sit amet nisl. Vivamus quis tortor vitae risus porta vehicula. See Section 2.

*Use footnote for providing further information about author (webpage, alternative address)—*not* for acknowledging funding agencies.

2.1 Headings: second level

Fusce mauris. Vestibulum luctus nibh at lectus. Sed bibendum, nulla a faucibus semper, leo velit ultricies tellus, ac venenatis arcu wisi vel nisl. Vestibulum diam. Aliquam pellentesque, augue quis sagittis posuere, turpis lacus congue quam, in hendrerit risus eros eget felis. Maecenas eget erat in sapien mattis porttitor. Vestibulum porttitor. Nulla facilisi. Sed a turpis eu lacus commodo facilisis. Morbi fringilla, wisi in dignissim interdum, justo lectus sagittis dui, et vehicula libero dui cursus dui. Mauris tempor ligula sed lacus. Duis cursus enim ut augue. Cras ac magna. Cras nulla. Nulla egestas. Curabitur a leo. Quisque egestas wisi eget nunc. Nam feugiat lacus vel est. Curabitur consectetuer.

$$\xi_{ij}(t) = P(x_t = i, x_{t+1} = j | y, v, w; \theta) = \frac{\alpha_i(t) a^{w_t}_{ij} \beta_j(t+1) b^{v_{t+1}}_j (y_{t+1})}{\sum^N_{i=1} \sum^N_{j=1} \alpha_i(t) a^{w_t}_{ij} \beta_j(t+1) b^{v_{t+1}}_j (y_{t+1})} \tag{1}$$

2.1.1 Headings: third level

Suspendisse vel felis. Ut lorem lorem, interdum eu, tincidunt sit amet, laoreet vitae, arcu. Aenean faucibus pede eu ante. Praesent enim elit, rutrum at, molestie non, nonummy vel, nisl. Ut lectus eros, malesuada sit amet, fermentum eu, sodales cursus, magna. Donec eu purus. Quisque vehicula, urna sed ultricies auctor, pede lorem egestas dui, et convallis elit erat sed nulla. Donec luctus. Curabitur et nunc. Aliquam dolor odio, commodo pretium, ultricies non, pharetra in, velit. Integer arcu est, nonummy in, fermentum faucibus, egestas vel, odio.

Paragraph Sed commodo posuere pede. Mauris ut est. Ut quis purus. Sed ac odio. Sed vehicula hendrerit sem. Duis non odio. Morbi ut dui. Sed accumsan risus eget odio. In hac habitasse platea dictumst. Pellentesque non elit. Fusce sed justo eu urna porta tincidunt. Mauris felis odio, sollicitudin sed, volutpat a, ornare ac, erat. Morbi quis dolor. Donec pellentesque, erat ac sagittis semper, nunc dui lobortis purus, quis congue purus metus ultricies tellus. Proin et quam. Class aptent taciti sociosqu ad litora torquent per conubia nostra, per inceptos hymenaeos. Praesent sapien turpis, fermentum vel, eleifend faucibus, vehicula eu, lacus.

3 Examples of citations, figures, tables, references

3.1 Citations

Citations use `natbib`. The documentation may be found at

`http://mirrors.ctan.org/macros/latex/contrib/natbib/natnotes.pdf`

Here is an example usage of the two main commands (`citet` and `citep`): Some people thought a thing [Kour and Saabne, 2014a, Hadash et al., 2018] but other people thought something else [Kour and Saabne, 2014b]. Many people have speculated that if we knew exactly why Kour and Saabne [2014b] thought this...

3.2 Figures

Suspendisse vitae elit. Aliquam arcu neque, ornare in, ullamcorper quis, commodo eu, libero. Fusce sagittis erat at erat tristique mollis. Maecenas sapien libero, molestie et, lobortis in, sodales eget, dui. Morbi ultrices rutrum lorem. Nam elementum ullamcorper leo. Morbi dui. Aliquam sagittis. Nunc placerat. Pellentesque tristique sodales est. Maecenas imperdiet lacinia velit. Cras non urna. Morbi eros pede, suscipit ac, varius vel, egestas non, eros. Praesent malesuada, diam id pretium elementum, eros sem dictum tortor, vel consectetuer odio sem sed wisi. See Figure 1. Here is how you add footnotes. [2] Sed feugiat. Cum sociis natoque penatibus et magnis dis parturient montes, nascetur ridiculus mus. Ut pellentesque augue sed urna. Vestibulum diam eros, fringilla et, consectetuer eu, nonummy id, sapien. Nullam at lectus. In sagittis ultrices mauris. Curabitur malesuada erat sit amet massa. Fusce blandit. Aliquam erat volutpat. Aliquam euismod. Aenean vel lectus. Nunc imperdiet justo nec dolor.

3.3 Tables

See awesome Table 1.

The documentation for `booktabs` ('Publication quality tables in LaTeX') is available from:

`https://www.ctan.org/pkg/booktabs`

[2] Sample of the first footnote.

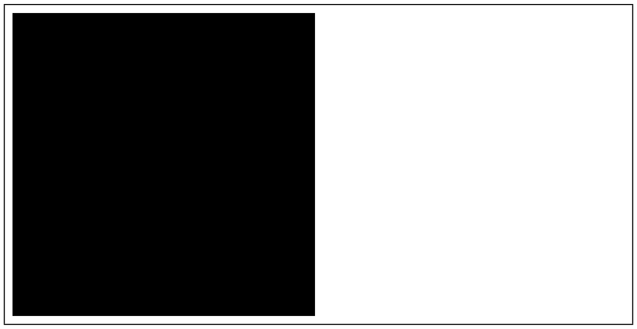

Figure 1: Sample figure caption.

Table 1: Sample table title

Part		
Name	Description	Size (μm)
Dendrite	Input terminal	$\sim$100
Axon	Output terminal	$\sim$10
Soma	Cell body	up to 10^6

3.4 Lists

- Lorem ipsum dolor sit amet
- consectetur adipiscing elit.
- Aliquam dignissim blandit est, in dictum tortor gravida eget. In ac rutrum magna.

References

George Kour and Raid Saabne. Real-time segmentation of on-line handwritten arabic script. In *Frontiers in Handwriting Recognition (ICFHR), 2014 14th International Conference on*, pages 417–422. IEEE, 2014a.

Guy Hadash, Einat Kermany, Boaz Carmeli, Ofer Lavi, George Kour, and Alon Jacovi. Estimate and replace: A novel approach to integrating deep neural networks with existing applications. *arXiv preprint arXiv:1804.09028*, 2018.

George Kour and Raid Saabne. Fast classification of handwritten on-line arabic characters. In *Soft Computing and Pattern Recognition (SoCPaR), 2014 6th International Conference of*, pages 312–318. IEEE, 2014b. doi:10.1109/SOCPAR.2014.7008025.
