# Supplementary figures and images for: Named entity recognition of pharmacokinetic parameters in the scientific literature
Source: Sci Rep. 2024 Oct 8;14:23485. doi: 10.1038/s41598-024-73338-3 (PMC11461509; doi:10.1038/s41598-024-73338-3)

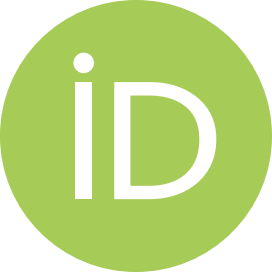

Supplement: Supplementary file 1 — Supplementary Information. [file 41598_2024_73338_MOESM1_ESM.zip › orcid.pdf]

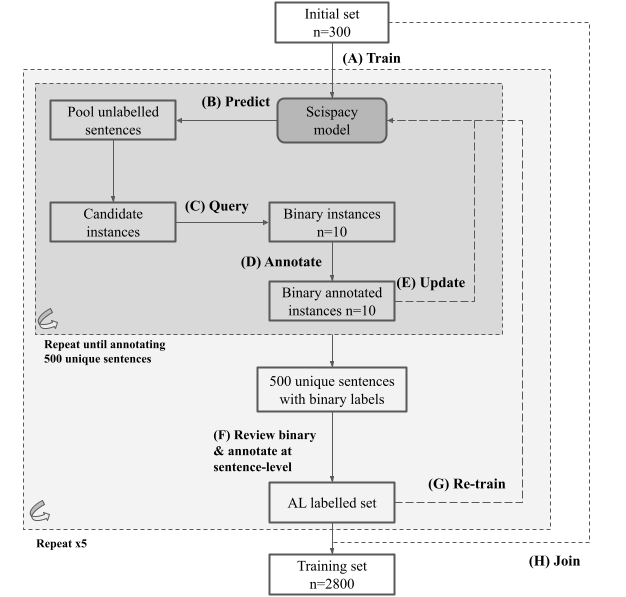

Supplement: Supplementary file 1 — Supplementary Information. [file 41598_2024_73338_MOESM1_ESM.zip › images/al_schema.png]

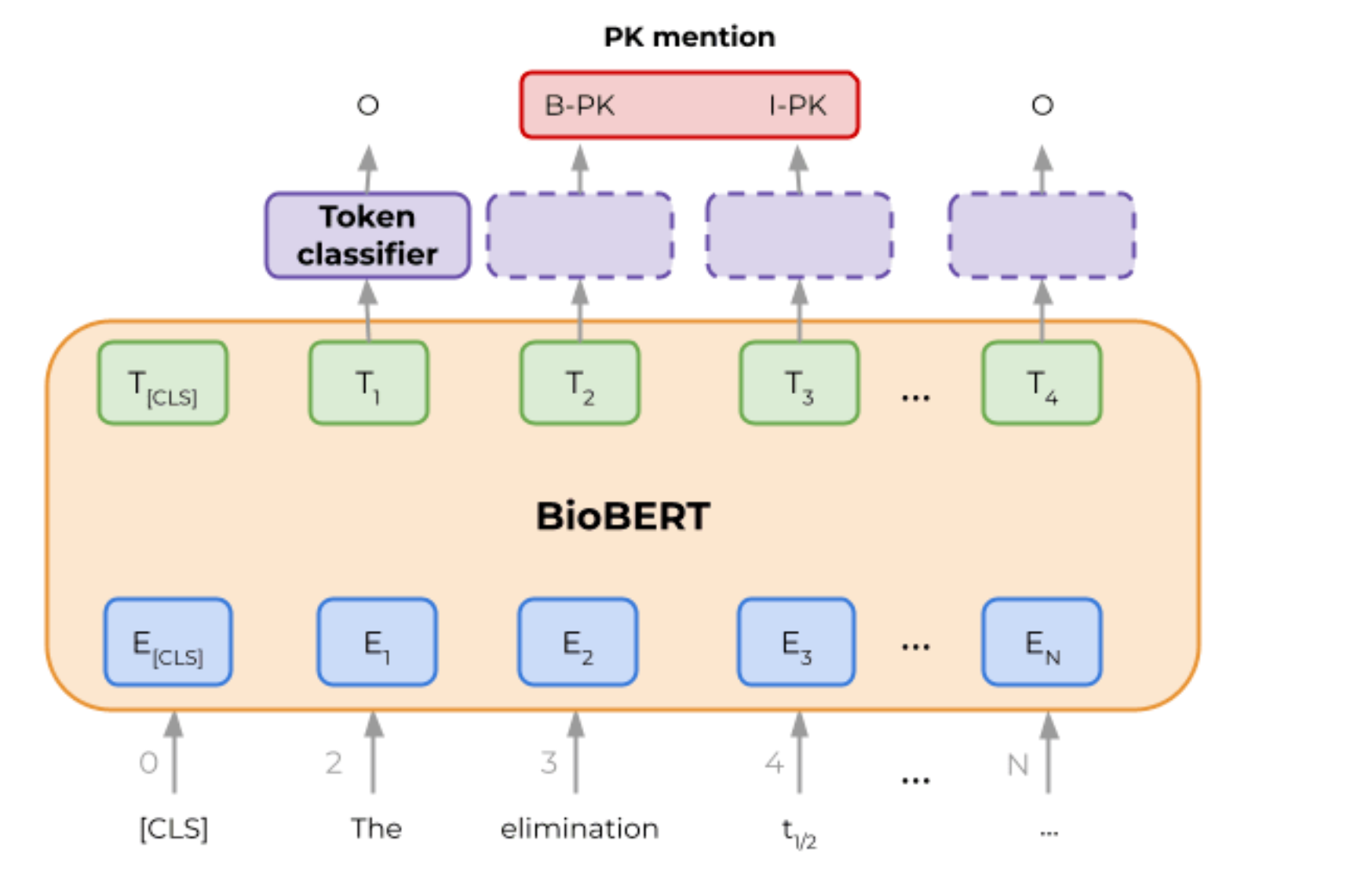

Supplement: Supplementary file 1 — Supplementary Information. [file 41598_2024_73338_MOESM1_ESM.zip › images/bert_architecture.png]

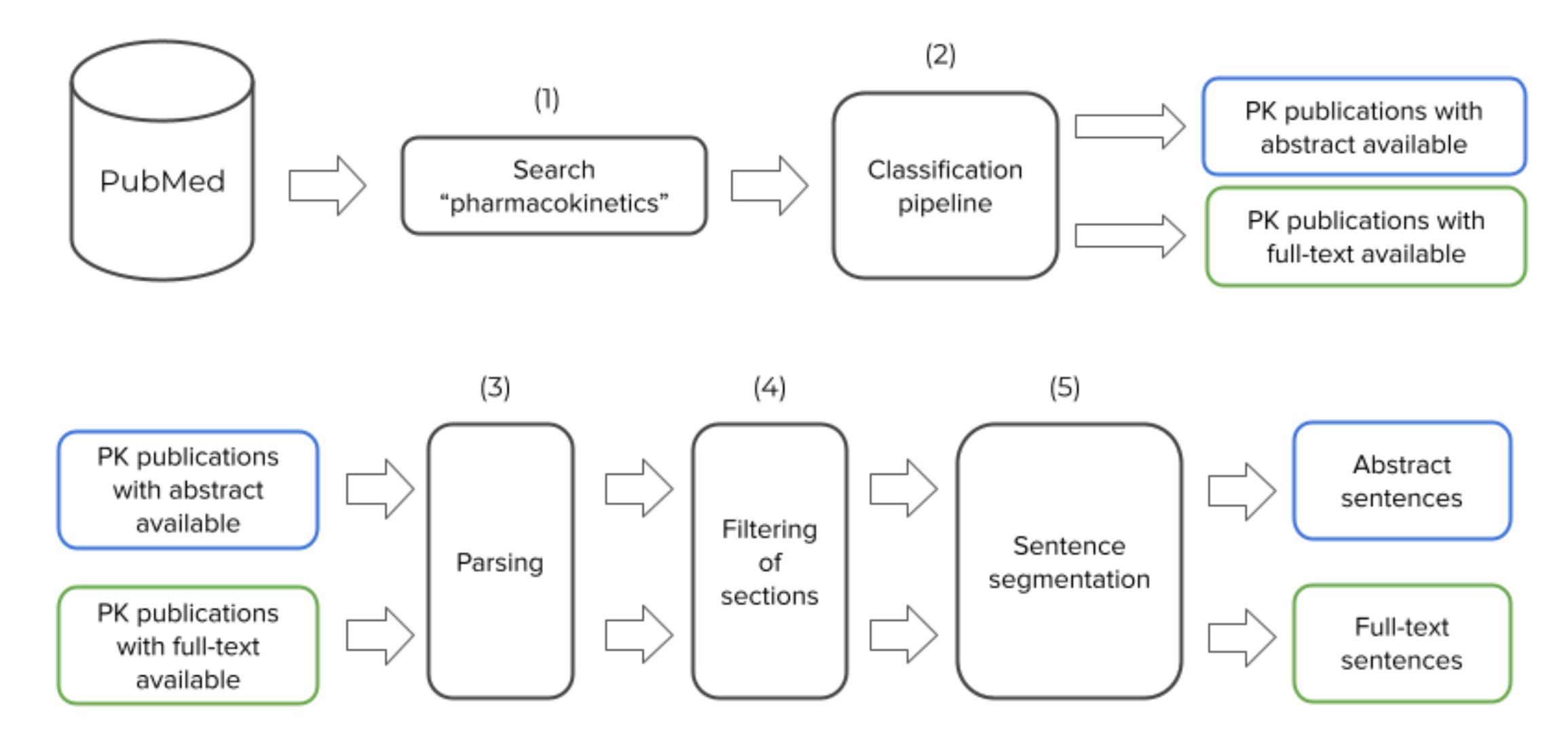

Supplement: Supplementary file 1 — Supplementary Information. [file 41598_2024_73338_MOESM1_ESM.zip › images/corpus_source.png]

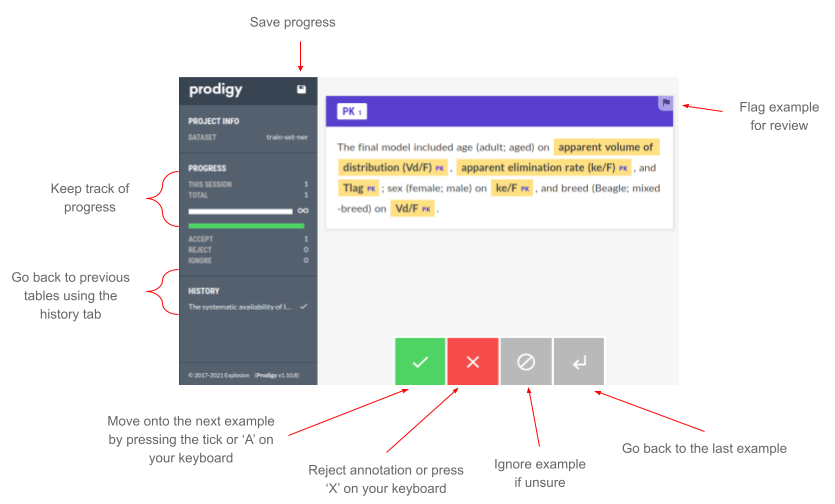

Supplement: Supplementary file 1 — Supplementary Information. [file 41598_2024_73338_MOESM1_ESM.zip › images/interfacener.png]

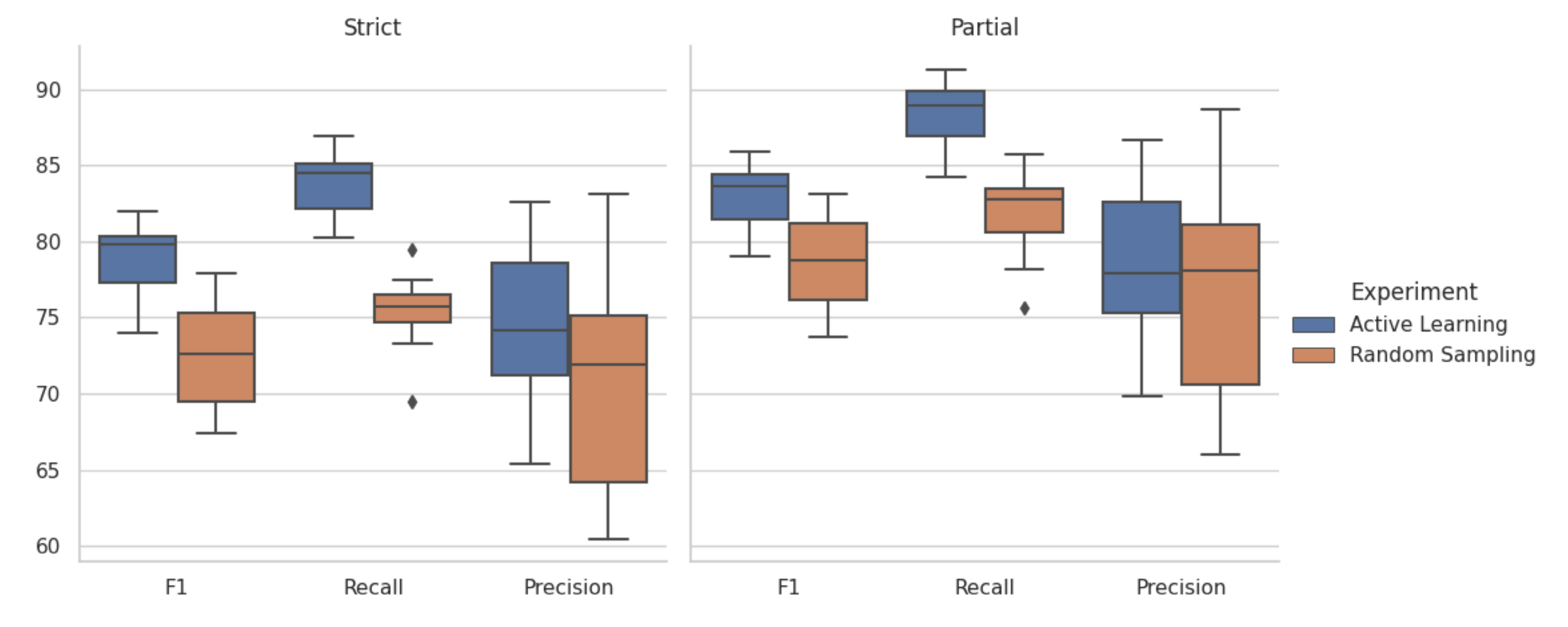

Supplement: Supplementary file 1 — Supplementary Information. [file 41598_2024_73338_MOESM1_ESM.zip › images/results_active_learning.png]
